# Preparation and Properties of Composite Double-Network Gel for Inhibiting Coal Spontaneous Combustion

**DOI:** 10.3390/molecules29143365

**Published:** 2024-07-17

**Authors:** Jianguo Wang, Zhenzhen Zhang, Wen Fu, Yifan Zhao

**Affiliations:** College of Safety Science and Engineering, Xi’an University of Science and Technology, Xi’an 710054, China; 15529066075@163.com (Z.Z.); 18392137326@163.com (W.F.);

**Keywords:** coal spontaneous combustion, composite inhibitor, coal molecular microstructure, gel performance

## Abstract

In order to improve the inhibition effect of gel on coal spontaneous combustion, a chitosan (CS)/polyacrylamide (PAM)/metal ion (Al_3_^+^) composite double-network gel was developed in this study. The optimum formula of the composite double-network gel was determined using orthogonal experimentation. The microstructure, water retention, compressibility, and anti-destruction properties of the composite double-network gel were analyzed. The inhibition effect of the composite double-network gel on coal spontaneous combustion was studied via infrared spectroscopy and a synchronous thermal analyzer from the micro and macro perspectives. The results show that the composite double-network gel has a denser interpenetrating double-network structure and a larger void ratio than the ordinary gel. The water retention rate was 55% after standing at 150 °C for 12 h. The deformation memory ratio of the composite double-network gel was 78%, which was 26.8% higher than that of the ordinary gel, and the compressive strength also increased by 59.96%. In addition, the critical temperature point and the maximum thermal weight-loss rate temperature point decreased by 7.01 °C and 39.62 °C, respectively, and the composite double-network gel effectively reduced active functional groups in the treated coal sample, such as hydroxyl and aliphatic hydrocarbons. In this study, a CS/PAM/Al_3_^+^ composite double-network gel was produced, which exhibited good gel performance and inhibition effects, with physical effects such as the covering, wetting, and cementation of coal.

## 1. Introduction

Coal spontaneous combustion, caused by heat accumulation within the coal mass, is one of the five major disasters in mines [1,2], posing a significant threat to mine safety. The toxic and harmful gases and high-temperature smoke released during coal fires can cause severe environmental pollution and endanger the lives of workers underground and in surrounding areas [3,4,5]. Coal spontaneous combustion involves oxygen molecules first forming physical and chemical adsorption heat on the surface of the coal, so that its temperature rises slowly. The increase in temperature enables the oxygen molecules to overcome the potential resistance, and deep oxidative decomposition reactions occur with the active functional groups on the surface of the coal molecules, producing small-molecule gases and releasing a large amount of reaction heat. This heat accumulates in the coal, eventually leading to spontaneous combustion. While existing fire prevention and extinguishing techniques such as grouting [6,7], inert gas injection, and foam application [8,9] have achieved some critical successes in preventing and controlling mine fires, there are still limitations in their application as mining depths and durations continue to increase. For instance, grouting techniques are prone to clogging pipelines, and as the grouting material dries out, it cracks and forms new air leakage channels on the coal surface [10]. Inert gas injection techniques require high sealing standards in practical applications, especially in areas with severe air leakage, which can lead to prolonged fire suppression times and the risk of re-ignition [11]. Foam-based methods also suffer from poor stability and easy dehydration and cracking [12]. Therefore, the development of effective fire prevention and extinguishing materials is crucial for the sustainable development of coal mining enterprises.

Dual-network gels have demonstrated good potential and application prospects in preventing and controlling coal spontaneous combustion [5]. Double-network gels have good water retention, high-temperature resistance, and coal spontaneous combustion inhibition characteristics, and their cost is low. In the prevention and control of coal spontaneous combustion, the characteristics of such gels can significantly reduce the risk of spontaneous combustion, protect the lives of miners, and reduce the loss of coal resources. Typically, dual-network gels consist of two distinct polymer networks: a rigid polymer network with a high cross-linking density as the first network and a flexible polymer network with a low density as the second network [13]. To further enhance the effectiveness and stability of dual-network gel technology, researchers have incorporated physical cross-linking interactions, such as hydrogen bonds, ionic bonds, electrostatic interactions, and hydrophobic associations, into gel systems to create fully physically cross-linked gels with both physical and chemical effects. These gels exhibit excellent self-healing and fatigue resistance [14]. For instance, Ye et al. [15] synthesized a konjac glucomannan/polyacrylamide dual-network gel system and studied the effects of covalent cross-linking, ionic cross-linking, and monomer ratio on the gel’s mechanical properties. The results showed that the dual-network gel possessed higher strength than the single-network gel. Chen et al. [16] prepared a tough hydrogel with a nano-network structure, demonstrating its significant potential. Chen et al. [17] also fabricated a tough polyacrylamide gel (HPAAm gel) with hydrophobic cross-linking and a dual-network polyacrylamide hydrogel (DN-HPAAm) containing hydrophobic cross-linking. The DN-HPAAm gel exhibited better compressive strength and cyclic compression performance compared to the HPAAm gel. Sun [18] incorporated an ion-crosslinked sodium alginate network, which replaced the traditional covalently cross-linked rigid network, into a polyacrylamide network to form a composite gel with a complex dual-network structure. This hydrogel exhibited better mechanical properties compared to single chemical cross-linking. Chen [6] employed free radical polymerization to obtain a high-strength and high-toughness polyacrylamide dual-network gel, which possessed excellent toughness and plasticity. These enhanced effects are closely related to the cross-linking effects within the gel. During stress application, the physical cross-linking interactions within the gel molecules rapidly break, consuming significant energy. The chemically cross-linked polyacrylamide provides high toughness to the entire network system, further enhancing the hydrogel’s mechanical strength. While dual-network structures have significantly improved the mechanical properties of gels, their structures can suffer permanent and irreversible damage due to the breakage of chemical bonds, which means they may not meet the long-term needs of coal fire prevention and extinguishing work [19]. The water retention and thermal stability of dual-network gels also have certain limitations.

Based on the above, this study involved introducing a metal ion cross-linking agent with high cross-linking density into a dual-network gel system to prepare a chitosan/polyacrylamide/metal ion composite dual-network fire prevention and extinguishing gel. This gel has a controllable gelation time, excellent strength, and resistance to damage, allowing it to encapsulate coal for extended periods to achieve fire prevention and extinguishing effects. Orthogonal experiments with four factors and three levels were designed, and significance analysis was performed to determine the optimal gel formula. The gel’s microstructure, thermal stability, mechanical properties, and coal spontaneous combustion inhibition characteristics were analyzed in detail.

## 2. Results and Discussion

### 2.1. Analysis of the Impact of Component Content on Gel Properties

Using gelation time, viscosity, and strength as indicators, a four-factor, three-level orthogonal experiment was conducted with chitosan (CS) content A, acrylamide (AM) content B, N, N′-methylenebisacrylamide (MBA) content C, and a blank control group D as factors. The level codes of each factor are shown in Table 1, and the experimental design and results are presented in Table 2.

(1) The effects of CS, AM, and MBA content on gelation time.

**Figure 1:**
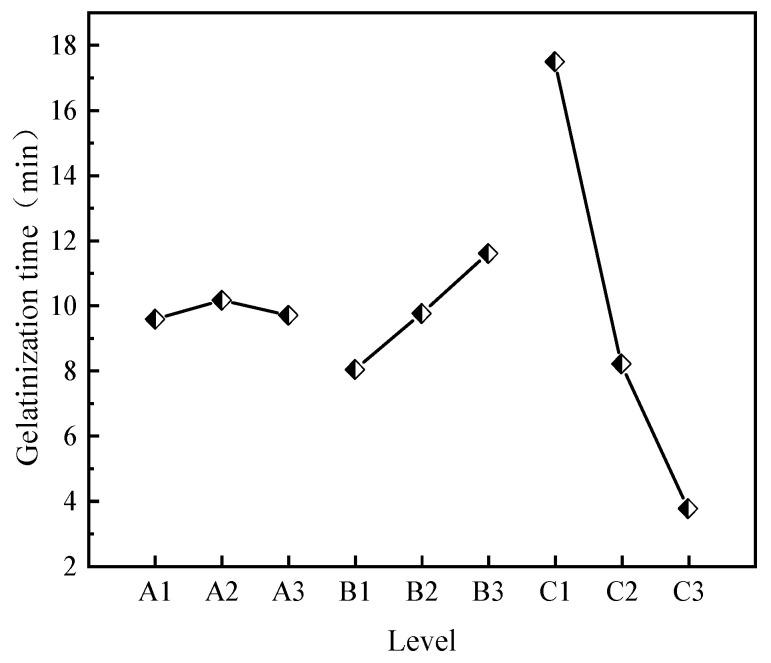
Effects of various factor levels on gelation time.

Statistical analysis was performed on the impact of different factors and levels on gelation time based on the orthogonal experiment results. The impact of each factor level on gelation time is shown in Figure 1. The significance analysis results of each factor are presented in Table 3.

From Table 3, it can be seen that the R^2^ value is 0.997, and the closer the R^2^ value is to 1, the closer the calculation results are to reality. MBA has the largest range, indicating that the change in MBA concentration has the strongest influence on gelation time. Gelation time is negatively correlated with MBA concentration, meaning that the higher the MBA content, the shorter the gelation time. AM concentration change is positively correlated with gelation time, indicating that the higher the AM content, the longer the gelation time. The impact of CS on gelation time increases first and then decreases with increasing dosage, showing no obvious pattern. The impact of the three factors on gelation time is MBA > AM > CS. The effect of CS content on gelation time is relatively small, and gelation time is mainly influenced by acrylamide (AM) and the cross-linking agent (MBA), with MBA being the primary factor. This also aligns with the above-mentioned mechanism analysis, where the first network composed of polyacrylamide molecular chains serves as the main network, so gelation time is primarily affected by MBA content; specifically, the greater the content of the crosslinker MBA, the faster the gel-forming time. Based on the analysis of the impact of various factors on gelation time in the orthogonal experiment, gels with different gelation times can be formulated for different coal spontaneous combustion depths, enhancing the applicability of gel fire prevention materials.

(2) The effects of CS, AM, and MBA content on viscosity.

**Figure 2:**
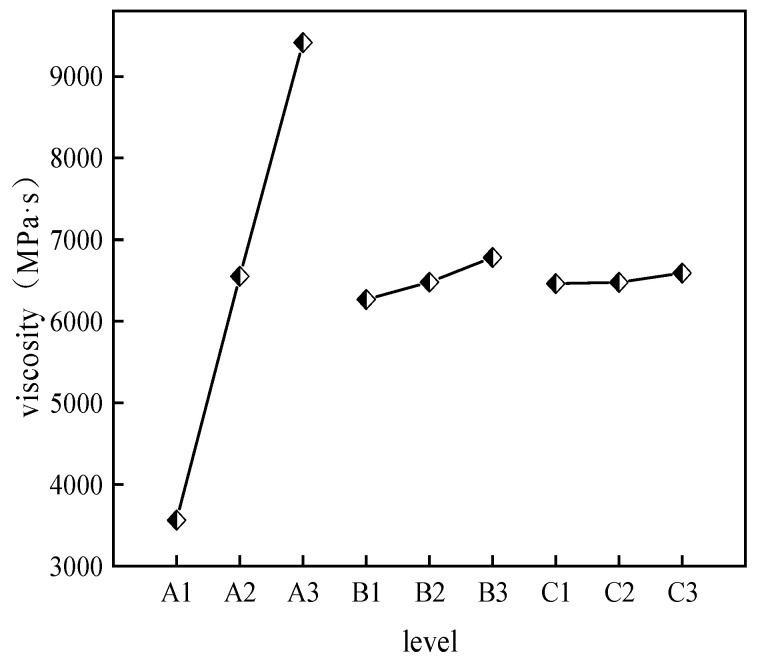
Effects of various factor levels on gel viscosity.

The impact of each factor level on gel viscosity is shown in Figure 2. The significance analysis results of each factor are presented in Table 4.

The R^2^ value is 0.999, indicating that the calculation results are in line with expectations. As can be seen from Table 4, all three factors have a positive correlation with gel viscosity, meaning that gel viscosity increases with increasing concentration. From the polar deviation in the figure, it can be seen that the polar deviation for CS is the largest, followed by AM, and MBA has the smallest deviation, indicating that the impact of the three factors on viscosity is CS > AM > MBA. The significance analysis results show that the *p* value of CS is less than 0.05, indicating that the change in CS content has a significant impact on gel viscosity. Specifically, the higher the CS content, the higher the gel viscosity. This is because CS is a natural polymer substance with many amino and hydroxyl functional groups in its molecular structure, which gives it a large viscosity correlation when forming a gel in water. Based on the analysis of the impact of various factors on gel viscosity in the orthogonal experiment, gel formulations with suitable viscosity for underground coal mines can be selected.

(3) Impact of CS, AM, and MBA Content on Strength

**Figure 3:**
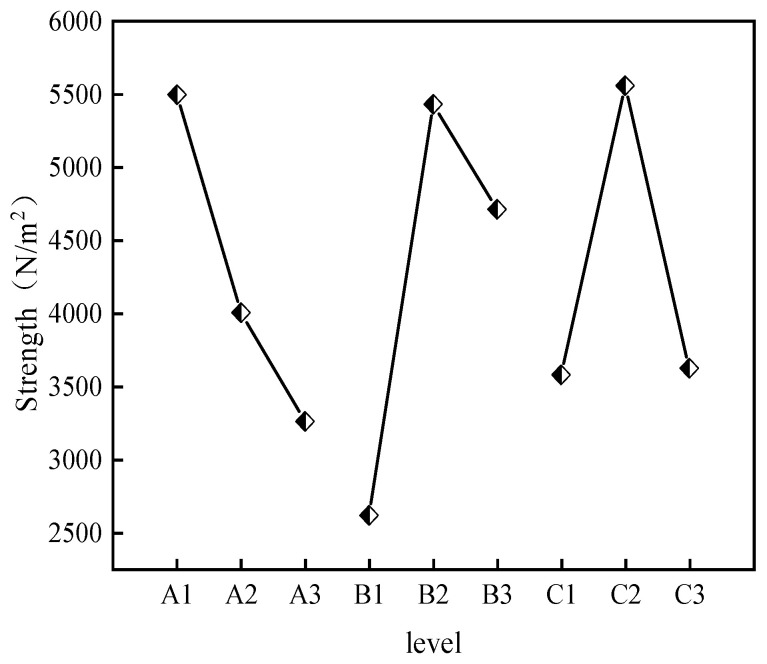
Effects of various factor levels on gel strength.

The impact of various factor levels on gel viscosity is shown in Figure 3, and the significance analysis results for each factor are presented in Table 5.

Using three-factor variance analysis to study the effects of CS, AM, and MBA on strength, it can be observed from Table 5 that the change in CS content is negatively correlated with gel strength, while the changes in AM and MBA contents show no obvious pattern in gel strength. The significance calculation results indicate that CS, AM, and MBA do not exhibit significance, suggesting that they do not have a differential relationship with strength. Because the two networks of the double-network gel are interspersed with each other to achieve toughening, their strength is not only influenced by the content of the two networks but also related to the crosslinking density and structure between them. Therefore, changes in the content of a single factor do not have a significant impact on the strength of the gel.

In summary, the changes in AM and MBA content have a significant impact on the gelation time of the gel. For effective application in the prevention and control of underground coal mine fires, the gelation time of the gel should not be too fast or too slow. An excessively fast gelation time will prevent the gel from penetrating into deep cracks in the coal seam, while an excessively slow gelation speed will result in the gel flowing out of the gaps and being unable to remain on the coal surface. The gelation time of the gel should be controlled to within 5 to 10 min. The content of CS has a significant impact on gel viscosity. To ensure the gel’s fluidity and permeability, the viscosity of the gel should be chosen within a moderate range. To better adapt to the prevention and control of spontaneous combustion fires in coal mines, the gel’s strength needs to be sufficiently high. Therefore, with the objectives of achieving appropriate gelation time and viscosity, as well as high gel strength, nine sets of orthogonal experiments were conducted. The fifth group, consisting of 2.5 wt% CS, 11 wt% AM, 0.6‰ MBA, and 2 wt% acetic acid solution, with the initiator APS and catalyst TEMED involved in the polymerization reaction, was ultimately selected as the optimal gel formula.

### 2.2. Microstructural Characterization of Double-Network Gel

**Figure 4:**
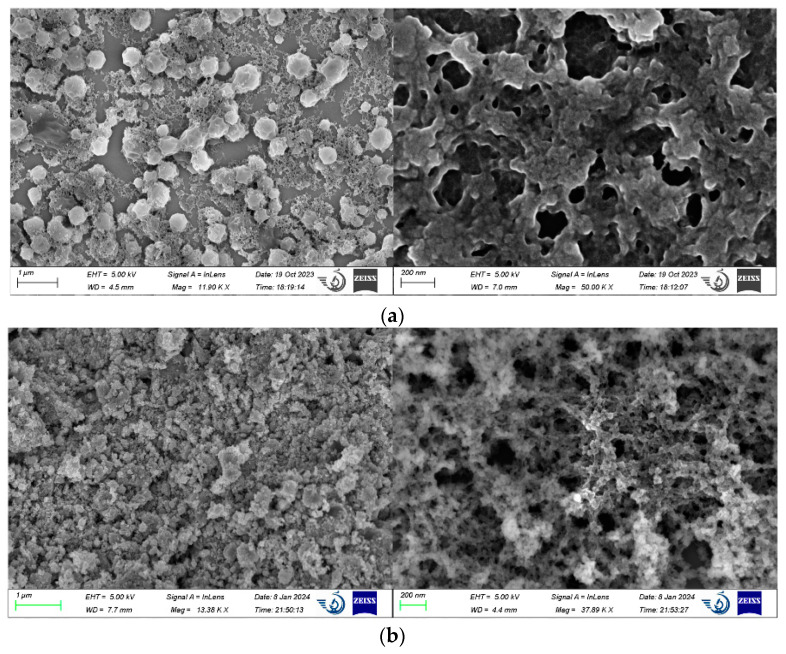
(**a**)**.** Scanning electron microscope images of plain gel. (**b**) Scanning electron microscope images of composite double-network gel.

Figure 4a shows the scanning electron microscope image of the plain gel. It can be observed that the dried plain gel has an interconnected internal structure, forming a dense interpenetrating double-network structure. This structure greatly improves the stability within the gel, enabling it to possess high strength and resist more pressure during the prevention of coal spontaneous combustion, thus enhancing its fire prevention and extinguishing effects. There are also a large number of pore structures within the gel, which significantly increase the gel’s contact area with water, enhancing its water absorption and water retention effects. After absorbing water, the gel covers the coal surface, and its uniform and dense structure enables it to isolate oxygen [20,21].

Figure 4b shows the scanning electron microscope image of the composite double-network gel. From the image, it can be seen that, compared to the plain gel, the composite double-network gel possesses a more densely arranged interpenetrating double-network structure and a larger pore ratio. This results in the composite double-network gel having higher strength and better water absorption effects. In the process of preventing coal spontaneous combustion, it can better moisten the coal surface, isolate oxygen, and reduce the risk of coal spontaneous combustion.

### 2.3. Water Retention Analysis

#### 2.3.1. Water Retention Analysis at Constant Temperature

**Figure 5:**
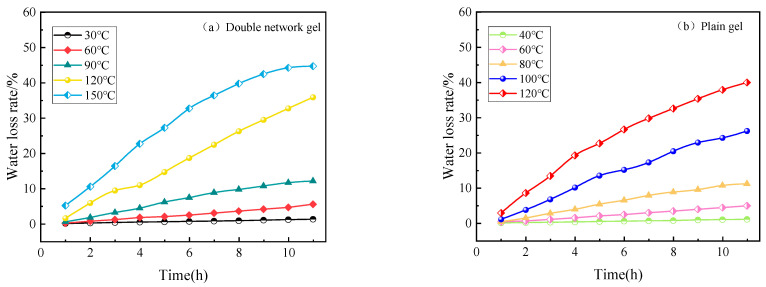
Water loss rate change curves of two gels under constant temperature conditions.

The stability of a gel directly affects its ability to inhibit coal spontaneous combustion [22]. It can be seen from Figure 5 that the water loss rates of the two gels gradually increase with time under different temperature conditions. Under 100 °C, the water loss rate of the two gels does not change much, the water loss is slow, and the water loss rate is basically maintained below 10%. When the temperature reaches 100 °C, the water loss rate begins to increase rapidly, and the water loss rate reaches about 40% within 12 h. Obviously, it can be seen that the composite double-network gel has a longer water retention time and better water retention.

**Figure 6:**
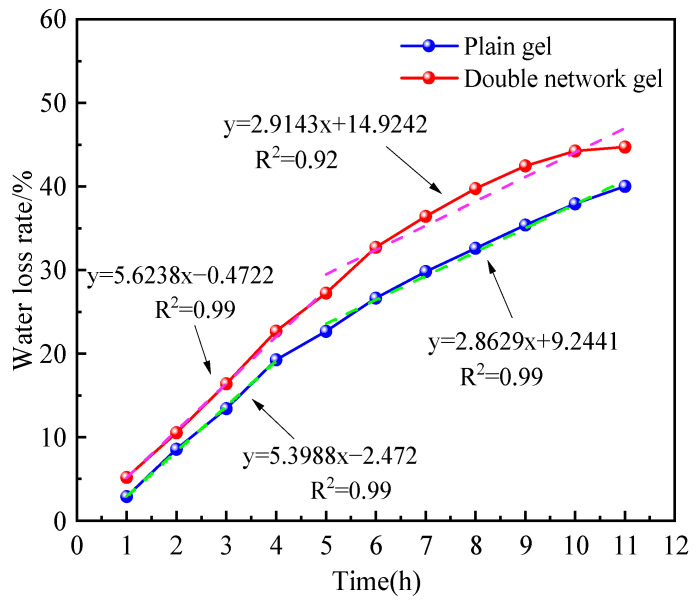
Fitting curves of water loss rates of two gels at 120 °C.

As shown in Figure 6, compared to the plain gel, the composite double-network gel exhibits a significant decrease in water loss rate under the same temperature conditions. After heating for 12 h, the water loss rate of the plain gel is 34.71%, while the water loss rate of the composite double-network gel is 40.1%, representing a 5.39% decrease. This indicates that the composite double-network gel has a better water retention effect. From the fitted curves, it can be observed that the water loss rate curves of both gels can be divided into two parts: a rapid water loss stage and a stable water loss stage. The plain gel undergoes a rapid water loss stage from 0 to 5 h, with a rapid increase in water loss rate, reaching 27.2% within 5 h. It then enters a stable water loss state from 5 to 12 h, with a water loss rate change of 17.5% and a significantly reduced water loss rate. The composite double-network gel exhibits a rapid water loss state from 0 to 4 h, with a water loss rate of 19.25% during this period. From 4 to 12 h, it enters a stable water loss stage, ultimately stabilizing at a water loss rate of 40%. This suggests that compared to the plain gel, the composite double-network gel can reduce the duration of the rapid dehydration stage, enter the stable water loss stage earlier, and achieve a significant reduction in water loss rate.

#### 2.3.2. Water Retention Analysis under Heating

**Figure 7:**
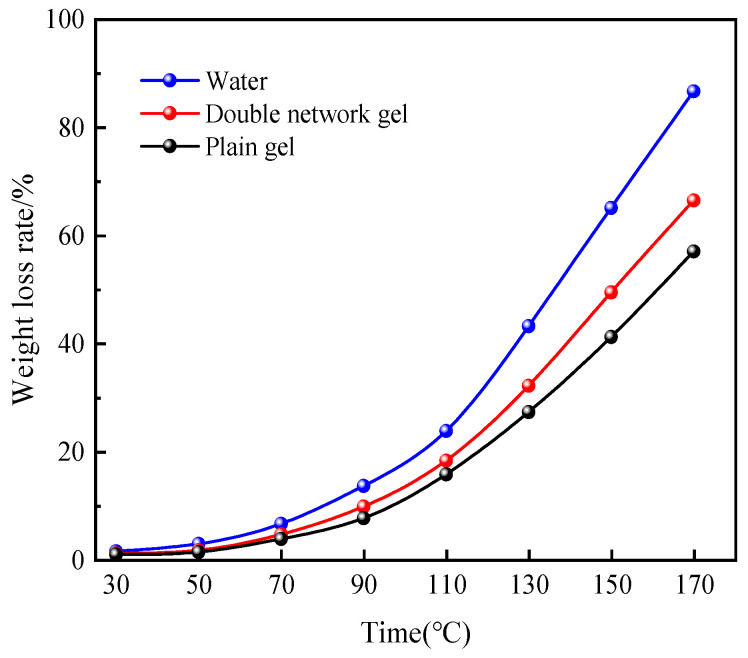
Weight-loss rate change curves under heating environment from 30 to ~170 °C.

As shown in Figure 7, water exhibits the highest weight-loss rate during the entire heating process, while both the plain gel and composite double-network gel have varying degrees of reduced weight-loss compared to water. The weight-loss rate of water reaches a maximum of 86.7% at 170 °C. Below 110 °C, the difference in weight-loss rate between the plain gel and the composite double-network gel is not significant. However, as the temperature increases, the difference in weight-loss rate between the two gels gradually increases. At 170 °C, the weight-loss rates reach 63.9% and 57.05%, respectively, representing reductions of 22.8% and 29.65% compared to the weight-loss rate of water. This indicates that both gels possess good thermal stability, and are able to lock in most of the water within the gel. Both gels maintain good thermal stability below 110 °C, but as the temperature increases, the composite double-network gel exhibits superior thermal stability.

### 2.4. Mechanical Property Analysis

Gel fire prevention and extinguishing materials are often applied to fractures on the surface of coal bodies. Apart from their flame-retardant effects, they also serve to fill and reinforce, seal leaks, and isolate oxygen. The double-network gel, due to its unique interpenetrating double-network structure, significantly improves the mechanical properties of the gel, reducing the damage caused by gravity-induced settlement and deformation of the coal body [23].

**Figure 8:**
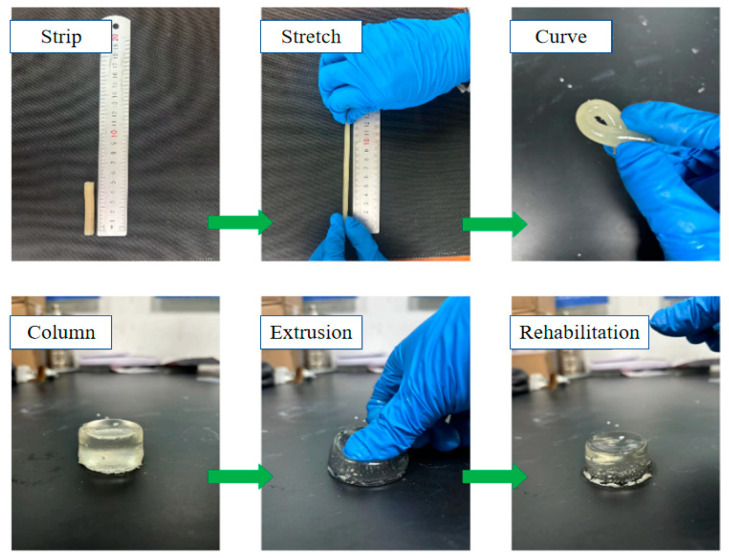
Digital images of gel under different mechanical actions.

Figure 8 demonstrates that the composite double-network gel possesses excellent mechanical strength and tensile properties, and is able to withstand large-scale stretching and bending, as well as recovering its original shape after undergoing high compressive deformation. When the fire prevention and extinguishing gel material is injected into the residual coal area of the goaf, it may be subjected to external forces such as roof collapse, leading to deformation or even damage to the material itself. Due to its unique interpenetrating double-network structure, the composite double-network gel exhibits excellent resistance to external deformation, enabling it to provide more durable coverage and encapsulation of residual coal in the goaf during gel fire prevention and extinguishing [24,25].

#### 2.4.1. Analysis of Resistance to Damage

**Figure 9:**
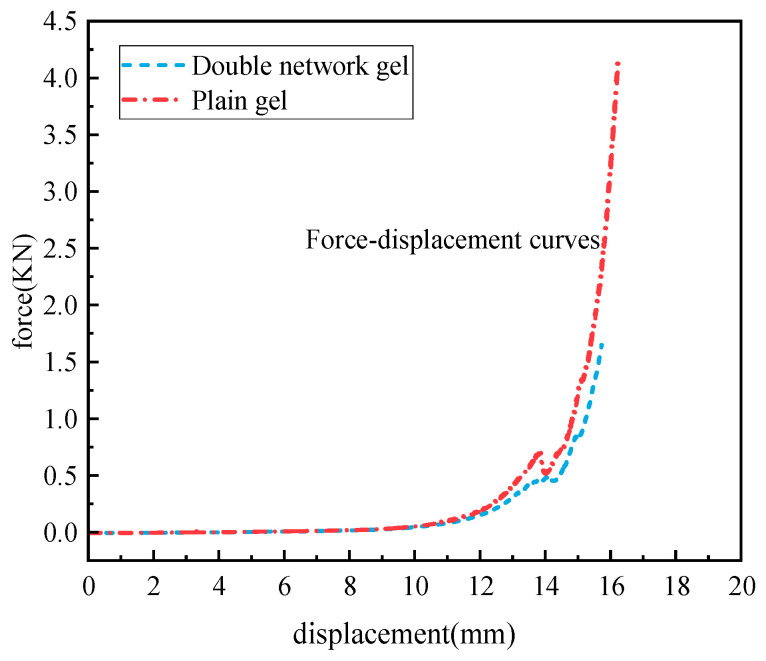
Force–displacement curves of two gels.

The analysis of anti-damage performance is an important parameter for gel fire prevention and extinguishing [26]. It reflects the gel’s ability to fill, reinforce, and isolate oxygen. The force–displacement curve is shown in Figure 9.

As can be seen from Figure 9, the force–displacement curves of the plain gel and the composite double-network gel exhibit a similar trend for the first 10 mm, which is essentially a smooth straight line. When the displacement exceeds 10 mm, the slope of the curve begins to change abruptly, and the force also increases with the increase in displacement. The force acting on the composite double-network gel is far greater than that acting on the plain gel. When the displacement of the plain gel reaches 15.7 mm, the bottom of the gel breaks, and upon removing the load from the upper part of the testing machine, the rebound height of the gel is 12.3 mm. For the composite double-network gel, when the displacement reaches 16.2 mm, the bottom of the gel breaks, and upon removing the load, the rebound height is 15.6 mm. After calculation, the deformation memory ratio of the composite double-network gel is 78%, while that of the plain gel is 61.5%, indicating that the composite double-network gel possesses excellent anti-damage performance and higher elasticity.

#### 2.4.2. Compression Performance Analysis

**Figure 10:**
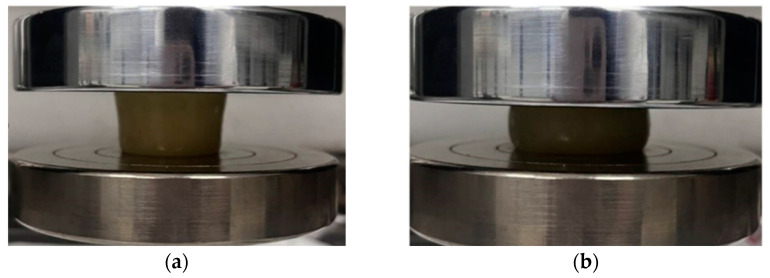
(**a**) Before gel compression performance test; (**b**) gel compression performance.

The compressive strength of the gel increases with the increase in strain. A CMT610 electronic universal testing machine was used to test the compression performance of the gel, and the stress–strain curves of the plain gel and the composite double-network gel are shown in Figure 10a,b.

**Figure 11:**
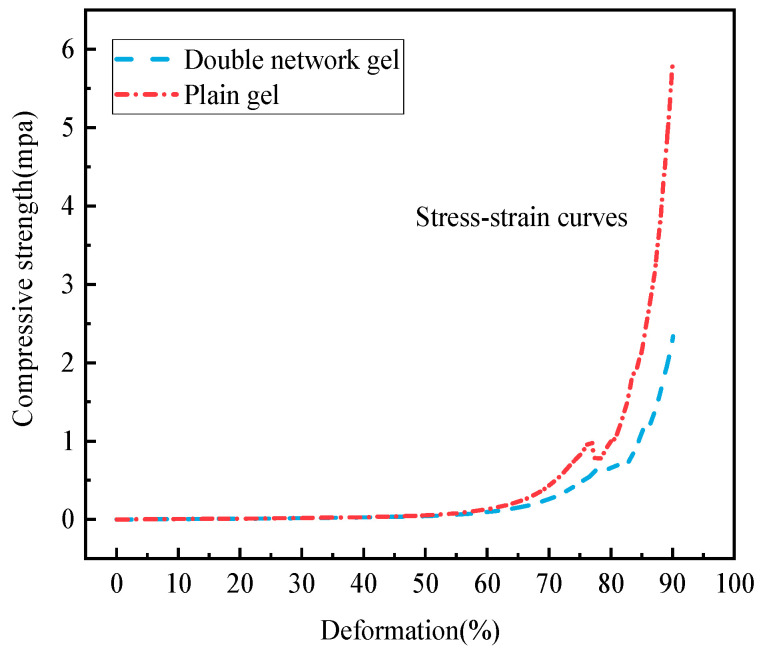
Stress–strain curves of two gels.

As can be seen from Figure 11, before the strain of both gels reaches 60%, the trend of the compressive strength change is basically the same. At this point, the pressure of the compressor has not caused substantial damage to the internal structure of the gel, and the slope of the curve is almost a smooth straight line, with little change in compressive strength, maintaining a level below 0.5 MPa. When the strain reaches 60%, the slope of the curve also changes abruptly, and the compressive strength begins to increase rapidly. It is evident from Figure 11 that the compressive strength of the composite double-network gel is far greater than that of the plain gel. When the strain of the composite double-network gel reaches 90%, the compressive strength reaches 5.92 MPa, while that of the plain gel reaches 2.37 MPa when its strain reaches 90%. The compressive strength of the composite double-network gel increases by 59.96% compared to the plain gel.

### 2.5. Analysis of the Characteristics of Inhibiting Coal Spontaneous Combustion

#### 2.5.1. Infrared Spectroscopy Analysis

**Figure 12:**
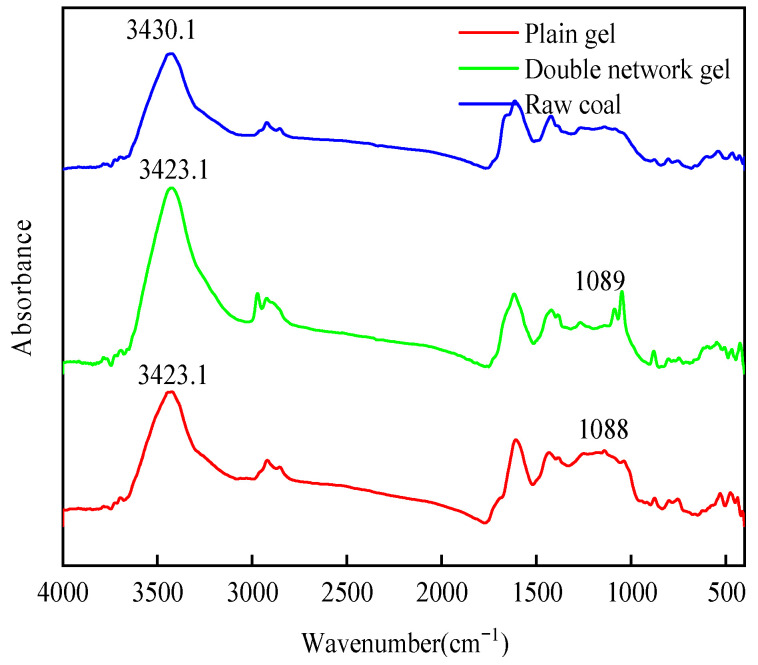
Infrared absorption spectra of different coal samples.

The infrared spectrum curve of raw coal molecules was measured experimentally. To ensure the smoothness of the curve at the data points, noise reduction was applied to the data using the Savitzky–Golay method [27], and the number of smoothing points was 15. The infrared spectra of three groups of samples are shown in Figure 12. From Figure 12, it can be seen that the spectral shapes of the three samples are roughly the same, with only slight differences in peak heights and positions. The plain gel group and the composite double-network gel group exhibited absorption peaks representing the polysaccharide structure (1089 cm^−1^), indicating that CS has successfully been incorporated into the hydrogel system. Compared to the raw coal, the hydroxyl absorption peak showed a significant shift (from 3430 to 3423 cm^−1^), indicating that new hydrogen bonds were formed between the amine groups on the polyacrylamide and the oxygen-containing functional groups on the CS chain, and the gel had formed a double-network structure through cross-linking.

**Figure 13:**
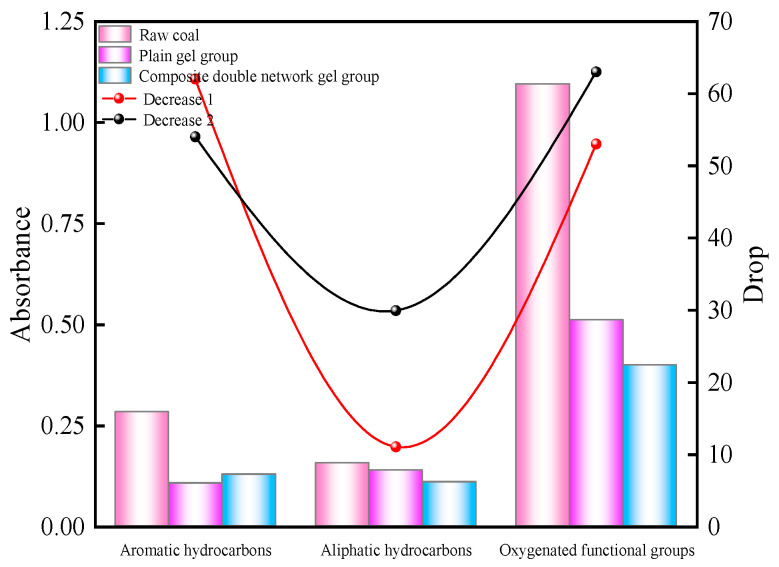
Absorbance change and its decline.

As can be seen from Figure 13, the absorbance of aromatic hydrocarbon C-H in the raw coal is 0.283, while the absorbances of C-H in the plain gel and composite double-network gel treated coal samples are 0.107 and 0.129, respectively, representing decreases of 62% and 54% compared to the raw coal. Both gel groups can significantly reduce the absorbance of C-H, specifically manifesting as a weakening of the out-of-plane bending vibration of C-H. The absorbance of aliphatic -CH_2_ and -CH_3_ in the raw coal is 0.157, while the gel-treated coal samples have absorbances of 0.139 and 0.11, respectively. The plain gel can increase the symmetric and asymmetric contractions of methyl and methylene groups, with a decrease of 18.6%. Due to the presence of Al_3_^+^, the composite double-network gel can induce an electron shift in the methyl and methylene groups, thus reducing their absorbance by 29.9%. The C-O absorbance of the raw coal is 0.546, while the gel groups have absorbances of 0.125 and 0.132. The total absorbance of -OH in the oxygen-containing functional groups of the raw coal is 0.549, with the in-plane bending vibration of hydroxyl being 0.177, the stretching vibration absorbances of alcoholic hydroxyl and phenolic hydroxyl being 0.336, and the free hydroxyl absorbance being 0.036. Neither the plain gel nor the composite gel showed a vibration peak with respect to free hydroxyl, indicating a significant decrease in free hydroxyl for the gels. The total hydroxyl absorbances of the two gel groups are 0.386 and 0.267, respectively, representing decreases of 29.6% and 51.3%. The composite double-network gel showed a greater decrease and more pronounced inhibitory effect.

**Figure 14:**
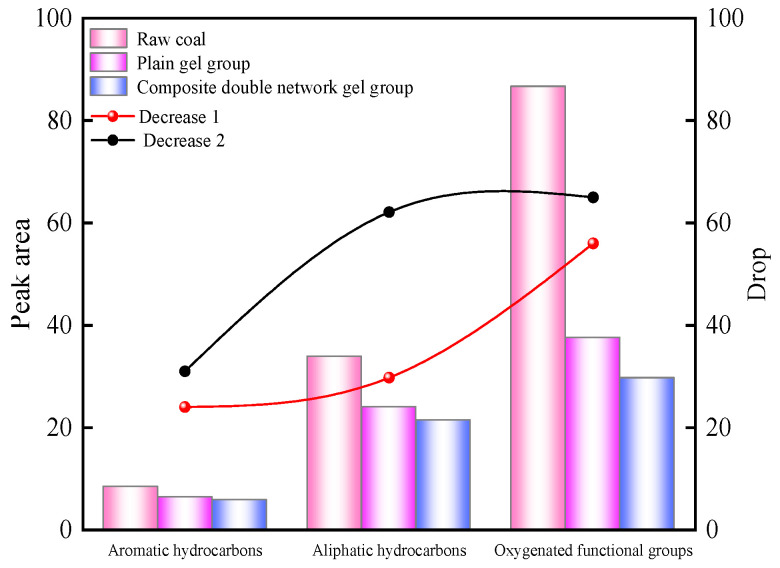
Peak area changes and decline.

As shown in Figure 14, the peak area of aromatic hydrocarbon C-H in the raw coal is 8.38, while the plain gel- and composite double-network gel-treated groups have peak areas of 6.32 and 5.76, respectively, representing decreases of 24% and 31%. The peak areas of methyl (-CH_3_) and methylene (-CH_2_) in the aliphatic hydrocarbons of the raw coal are both 33.83, while the gel-treated groups have peak areas of 23.96 and 21.73, representing decreases of 29% and 35%, respectively. The total peak area of oxygen-containing functional groups in the raw coal is 86.64, while the gel-treated groups have peak areas of 37.52 and 29.64, representing decreases of 56% and 65%, respectively. Among them, the peak area of hydroxyl (-OH) in the oxygen-containing functional groups of the raw coal is 44.52, while the gel-treated groups have peak areas of 33.9 and 33.4, representing decreases of 23% and 24.9%, respectively. The peak area of C-O in the raw coal is 42.12, while the gel-treated groups have peak areas of 13.56 and 8.27, representing decreases of 67% and 80%, respectively. From the above data, it can be seen that the composite double-network gel has the greatest inhibitory effect on the active oxygen-containing functional groups in the coal molecules.

Overall, both sets of gels have varying degrees of inhibitory effects on aromatic hydrocarbons, aliphatic hydrocarbons, and oxygen-containing functional groups in raw coal. The decreases in the absorbance and peak area of the coal samples treated with the composite double-network gel are significantly superior to those of the plain gel treatment group, indicating that the addition of Al_3_^+^ can more effectively induce the electron shift of functional groups, leading to the breaking of covalent bonds. By destroying the functional groups closely related to coal oxidation, the chemical inertness of coal molecules is induced, making it more difficult for the coal oxidation reaction to proceed and thus inhibiting coal spontaneous combustion.

#### 2.5.2. Thermogravimetry

**Figure 15:**
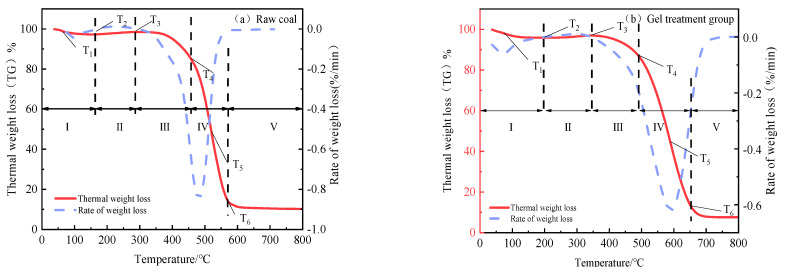
TG–DTG curves of raw coal and composite double-network gel-treated coal samples.

The TG–DTG curves of raw coal and the composite double-network gel treatment during the combustion process are shown in Figure 15, and the characteristic temperature points are listed in Table 6. T_1_ is the critical temperature, T_2_ is the pyrolysis temperature, T_3_ is the thermal decomposition temperature, T_4_ is the ignition temperature, T_5_ is the maximum weight-loss rate temperature, and T_6_ is the burnout temperature.

According to the variation of coal sample mass combined with the data in Table 6, the combustion process of the coal samples can be divided into five stages. Stage I is the initial weight-loss stage (0 °C~T_2_), where the mass of the coal sample gradually decreases due to water evaporation and gas desorption. Compared with raw coal, the critical temperature T1 and cracking temperature T_2_ of the gel-treated coal samples are delayed by 7.01 °C and 39.62 °C, respectively, because the polymer gel can lock in the moisture in coal, slow down water evaporation, and thus lower the temperature of the coal body. Stage II is the oxygen absorption and weight gain stage (T_2_~T_3_), where the coal reacts chemically with oxygen to produce oxidation products, and the oxidation generation rate is greater than the thermal decomposition rate, breaking the original dynamic balance of mass, with a slight increase in the curve. The maximum weight temperature T_3_ is shifted back by 55.58 °C compared to raw coal. Stage III is the thermal decomposition stage (T_3_~T_4_), where the ignition point temperature T_4_ is shifted back by 31.39 °C compared to raw coal, indicating that the metal ion composite double-network gel can effectively inhibit the process of thermal decomposition and delay the start of coal spontaneous combustion. Stage IV is the combustion stage (T_4_~T_6_), where intense chemical reactions occur in the combustible substances in the coal sample, producing large amounts of gas-phase products, and the TG curve drops rapidly. In this stage, the mass change rate of the gel treatment group is 48.46%, and the maximum weight loss rate temperature point T_5_ is shifted back by 63.18 °C compared to raw coal, while the burnout temperature T_6_ is shifted back by 85.57 °C. Stage V is the burnout stage (T_6_~800 °C), where the combustion reaction ends and the total mass of the coal sample changes little.

## 3. Materials and Methods

### 3.1. Experimental Materials and Equipment

The raw materials used include chitosan (CS, deacetylation degree ≥ 95%), glacial acetic acid (CH_3_COOH, 99%), N, N′-methylenebisacrylamide (MBA, 99%), acrylamide (AM, 99%), ammonium persulfate (APS), tetramethylethylenediamine (TEMED), and aluminum chloride (AlCl_3_). 

Chemicals were purchased from Shanghai Yien Chemical Technology Co., Ltd. (Shanghai, China). An LC-202-00 vacuum drying box and NDJ-8S digital viscosity meter were purchased from Lichen Technology Co., Ltd. (Lichen, China), a WDF-30 digital push-pull meter was purchased from Weidu Electronics Co., Ltd. (Weidu, China), and a JB-80SH digital top-mounted electronic stirrer was purchased from Xiniu Technology Co., Ltd. (Xiniu, China). The electron microscope was purchased from Hitachi Manufacturing Institute (Tokyo, Japan). Electronic universal testing machine was purchased from Jinan Hengsi Shengda Instrument (Jinan, China).

### 3.2. Sample Preparation Figures

**Figure 16:**
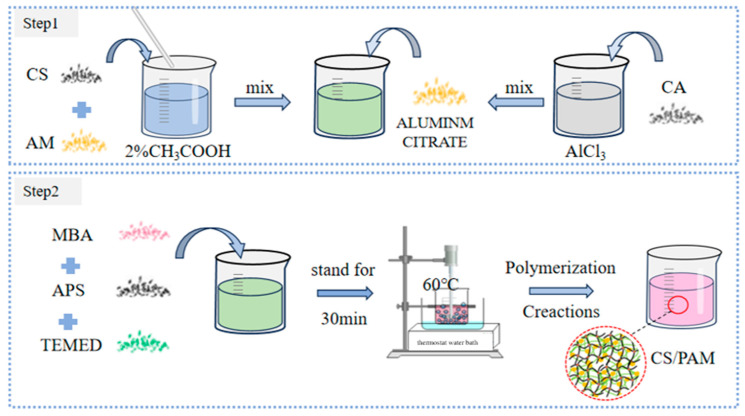
Preparation process.

**Figure 17:**
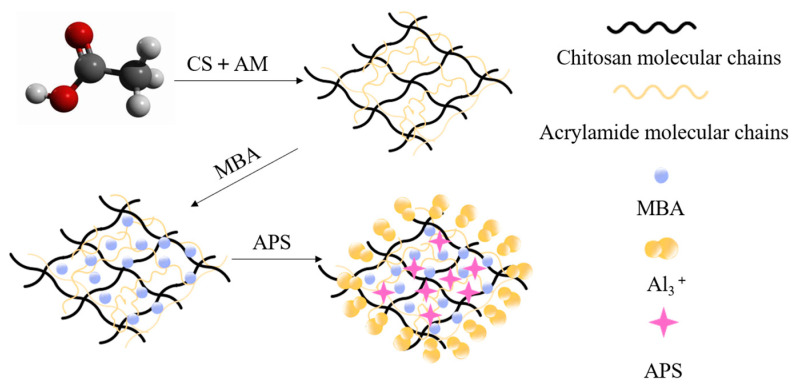
Diagram of gel formation mechanism.

In order to analyze the superior performance of the developed chitosan/polyacrylamide/metal ion composite double-network gel, a chitosan/polyacrylamide hydrogel was prepared. The preparation process is shown in Figure 16. The gel formation mechanism is shown in Figure 17.

A mixed solution of 40 mL was prepared. Firstly, 2% acetic acid was added, and then 2.5% CS and 11% AM were added in turn. At the same time, AlCl_3_ and citric acid were mixed at a ratio of 1:2 to prepare aluminum citrate. Next, the two solutions were mixed evenly, and then 0.6% MBA and small amounts of APS and TEMED were added and stirred until they had dissolved. After standing for 30 min, they were placed in a constant-temperature water bath at 60 °C for 1 h to prepare the chitosan/polyacrylamide water/metal ion composite double-network gel.

### 3.3. Study of Basic Properties of Double-Network Gel

#### 3.3.1. Microstructure Characterization of Double-Network Gel

An electron microscope (Gemini SEM500) was utilized to observe the microscopic morphology of the gel. Sample 1 (CS/PAM gel) was placed in a constant-temperature vacuum-drying oven after complete gelation and dried continuously at 35 °C until the gel was completely dehydrated. Sample 2 was the composite double-network gel with AlCl_3_ added, which underwent drying treatment under the same temperature conditions. Gold sputtering was performed on the crushed samples to obtain clearer images [28].

#### 3.3.2. Water Retention Test of Double-Network Gel

(1) Water retention test under constant temperature conditions

A total of 100 g of each of the two gels was prepared according to the optimized formula, after which they were placed in a blast-drying oven for continuous heating for 12 h, with the oven temperature set to 40 °C, 60 °C, 80 °C, and 100 °C. The gel mass was measured every 1 h during the test and the water loss rate of the gel was calculated using the following formula.

(1) $$W_{L}=\frac{m_{0}-m_{i}}{m_{0}}\times 100\%$$

where $W_{L}$ is the water loss rate, %; $m_{0}$ is the initial gel mass, g; $m_{i}$ is the gel mass at a certain time, g.

(2) Water retention test under heating conditions

An amount of 100 g of each of the two gels and water was placed in a constant-temperature drying oven, with the temperature increased by 10 °C every 30 min, and the weight change recorded at that moment.

#### 3.3.3. Mechanical Property Testing

A CMT610 electronic universal testing machine was used to test the compressive properties and anti-destructive properties of the gel, and changes in the gel height before and after the pressure test were recorded to determine the anti-destructive properties of the gel. All the tests were carried out at room temperature, and the experimental process is shown in Figure 10. The load unit of the compression experiment was 1 kN, and the compression loading rate was 1 mm/min. Each sample was tested three times in parallel to reduce the error of the experiment. The maximum deformation of the gel was 90%. The compressed sample was cylindrical with a diameter of 30 mm and a height of 10 mm. The calculation formula of the compressive strength is as follows.

(2) $$\sigma_{c}=F_{c}/A_{0}$$

In the formula, $F_{c}$ is the maximum load force when the sample compression variable is 90%, and $A_{0}$ is the original cross-sectional area of the sample.

### 3.4. Characteristics of Suppressing Low-Temperature Oxidation of Coal

#### 3.4.1. Infrared Spectroscopy Test

A Fourier-transform infrared spectrometer was used. The raw coal was mixed with water, double-network gel, and composite double-network gel at a ratio of 1:1, and then fully reacted at room temperature for 24 h. After drying, the sample was mixed with KBr solid particles at a ratio of 1:100, stirred evenly, and under a pressure of 8~10 MPa, pressed into a thin sheet with a thickness of 1 mm. The experimental test wave number range was 4000–400 cm^−1^, and the number of sample scans was 32.

#### 3.4.2. TGA

Characteristic temperature is one of the key indicators used to measure the spontaneous combustion process of coal [29]. Using a TGA/DSCI synchronous thermal analyzer (TG-DSCO1), the raw coal and gel-treated coal samples were placed in a constant-temperature drying oven set at 35 °C, dried for 24 h, then ground and passed through a 100-mesh sieve. The test was carried out in a nitrogen atmosphere, with a flow rate of 50 mL/min, a temperature range of 30 °C~800 °C, and a heating rate of 5 K/min.

## 4. Conclusions

In this study, a chitosan/polyacrylamide water/metal ion composite double-network gel was prepared, and its microscopic morphology, thermal stability, mechanical properties, and inhibition of coal spontaneous combustion were studied. The following conclusions were drawn:

(1) The ratio of the composite double-network gel was determined. The final gel formula was selected as 2.5 wt% CS, 11 wt% AM, 0.6‰ MBA, and 2 wt% acetic acid solution, with the initiator APS and the catalyst TEMED participating in the polymerization reaction.
(2) The chitosan/polyacrylamide water/metal ion composite double-network gel has good water retention and coal spontaneous combustion inhibition properties. The gel forms a smooth and dense film on the surface of the coal body, which can encapsulate the coal body and lock in water, reducing the water loss rate by 10%. Compared with raw coal, the composite double-network gel treatment group exhibited an increase in structures representing polysaccharides, as well as a shift in hydrogen bonds, demonstrating the formation of the double-network structure. In addition, the content of -OH and aliphatic hydrocarbon active functional groups in the coal molecules decreased by 24.9% and 31%, respectively, compared to raw coal. As the temperature increased, the characteristic temperature points for coal spontaneous combustion were delayed.
(3) The chitosan/polyacrylamide water/metal ion composite double-network gel has good anti-destruction and compression properties. The deformation memory of the composite double-network gel decreased by 6.5% compared to the plain gel, and the compressive strength of the composite double-network gel increased by 59.96% compared to the plain gel.
(4) In this work, only the fire prevention and extinguishing of a single coal type were studied. In order to improve the universality of the gel in the prevention and control of coal spontaneous combustion, different types of coal will be studied in future research.

## Figures and Tables

**Table 1:** Orthogonal test factor level table.

Level	Factor			
	A	B	C	D
	CS Content/%	AM Content/%	MBA Content/‰	Blank Group
1	2	10	0.4	1
2	2.5	11	0.6	2
3	3	12	0.8	3

**Table 2:** Orthogonal experiment results.

Experiment Number	CS Content (%)	AM Content (%)	MBA Content (%)	Gelatinization Time (min)	Viscosity (mPa·s)	Strength (N/m^2^)
1	2	10	0.4	15	3329	3423
2	2	11	0.8	3.75	3511	5225
3	2	12	0.6	10	3853	7846
4	2.5	10	0.8	2.5	6440	2320
5	2.5	11	0.6	8	6541	6726
6	2.5	12	0.4	20	6673	2968
7	3	10	0.6	6.6	9037	2105
8	3	11	0.4	17.5	9385	4346
9	3	12	0.8	5	9825	3326

**Table 3:** Significance analysis of CS, AM, and MBA content on gelation time.

Source of Differences	Sum of Squares of Dispersion	Degree of Freedom	Mean Squared	F	*p*
CS	0.572	2	0.286	0.533	0.652
AM	19.822	2	9.911	18.496	0.051
MBA	295.355	2	147.677	275.603	0.004

R^2^ = 0.997 *p* < 0.05 Significant.

**Table 4:** Significance analysis of CS, AM, and MBA content on viscosity.

Source of Differences	Sum of Squares of Dispersion	Degree of Freedom	Mean Squared	F	*p*
CS	513.646	2	256.823	106.5	0.001
AM	40.228	2	20.114	8.346	0.107
MBA	3.025	2	1.512	0.628	0.614

R^2^ = 0.999; *p* < 0.05 Significant.

**Table 5:** Significance analysis of CS, AM, and MBA content on strength.

Source of Differences	Sum of Squares of Dispersion	Degree of Freedom	Mean Squared	F	*p*
CS	77.991	2	98.995	2.427	0.292
AM	128.475	2	64.237	3.997	0.200
MBA	76.679	2	38.339	2.386	0.295

R^2^ = 0.898.; *p* < 0.05 Significant.

**Table 6:** Characteristic temperatures of raw coal and treated coal samples.

Coal Samples	T_1_ (°C)	T_2_ (°C)	T_3_ (°C)	T_4_ (°C)	T_5_ (°C)	T_6_ (°C)
Raw coal	62.04	160.77	287.89	454.08	525.16	564.67
Composite double-network gel treatment group	69.05	200.39	343.47	485.47	588.34	650.24

## Data Availability

Data are contained within the article.
